# 939. Embedding Palliative Care Triggers in Burn ICU Practice: A Quality Improvement Initiative

**DOI:** 10.1093/jbcr/irag033.506

**Published:** 2026-04-06

**Authors:** Kristin E Friedl, Meaghan Trainor, Lauren B Nosanov, Lori Mickelson, Angela Gibson

**Affiliations:** University of Wisconsin Burn and Wound Center, Madison, WI; University of Wisconsin Burn and Wound Center, Madison, WI; Emory University School of Medicine, Atlanta, GA; University of Wisconsin Burn and Wound Center, Madison, WI; University of Wisconsin Burn and Wound Center, Madison, WI

## Abstract

**Introduction:**

Patients admitted to burn ICUs with severe injuries and high rBaux scores face high mortality and complex decision-making needs. Despite evidence that early subspecialty palliative care (PC) involvement improves outcomes, consults are inconsistently initiated in this setting. A multidisciplinary team at a regional burn center conducted a quality improvement (QI) project to increase timely palliative care consults using a structured trigger based on rBaux score and comorbidities.

**Methods:**

From March to August 2024, the team implemented a PC consult trigger embedded into the burn ICU history and physical and tertiary burn assessment documentation. The trigger targeted patients with high rBaux scores (>80) and significant comorbidities (Charlson Comorbidity Index >5–7). The intervention included staff education, workflow integration, and nursing feedback surveys administered pre- and post-intervention. Data on PC consult frequency, provider behavior, and staff perceptions were tracked.

**Results:**

Prior to the intervention period, chart review on all patients with burn injuries over 20% TBSA showed that, among 8 total patients, none received a PC consult. During the intervention period, 8 patients met large burn criteria; 2 met full trigger criteria. One received a PC consult; the other transitioned to comfort care without consult. While overall consult rates did not increase significantly, provider documentation showed increased consideration of PC, with 6 of 8 patients having PC discussed in their initial evaluations. Nursing perceptions also shifted: pre-intervention, 88% of surveyed nurses felt PC was underutilized; post-intervention, only 44% felt so, and 66% reported satisfaction with the current consult rate. Staff also cited increased clarity and consistency in addressing goals of care.

**Conclusions:**

Embedding triggers for consideration of palliative care consultation resulted in a greater awareness among burn team providers of the potential for palliative care to benefit patients. It also led to greater nursing satisfaction with the frequency with which palliative care consultation was discussed or utilized.

**Applicability of Research to Practice:**

This initiative demonstrates that embedding structured PC consult triggers in burn ICU documentation can raise provider awareness and improve interdisciplinary alignment, even without a marked rise in consult numbers. Improved team communication and nurse satisfaction suggest value in maintaining the trigger system. Future directions include refining criteria and identifying the domains of primary palliative care provided by surgical teams and secondary palliative care provided by Palliative care consultant teams.

**Funding for the study:**

N/A.

